# Pattern of recurrence and overall survival in esophagogastric cancer after perioperative FLOT and clinical outcomes in MSI-H population: the PROSECCO Study

**DOI:** 10.1007/s00432-023-04636-y

**Published:** 2023-02-16

**Authors:** Floriana Nappo, Lorenzo Fornaro, Luca Pompella, Silvia Catanese, Daniele Lavacchi, Andrea Spallanzani, Alessandro Cappetta, Marco Puzzoni, Sabina Murgioni, Giulia Barsotti, Giuseppe Tirino, Antonio Pellino, Caterina Vivaldi, Antonia Strippoli, Giuseppe Aprile, Samantha Di Donato, Elena Mazza, Michele Prisciandaro, Lorenzo Antonuzzo, Vittorina Zagonel, Stefano Cascinu, Ferdinando De Vita, Sara Lonardi

**Affiliations:** 1 Medical Oncology 1, Veneto Institute of Oncology IOV-IRCCS, Via Gattamelata 64, 35128 Padua, Italy; 2 Medical Oncology, Azienda Ospedaliero-Universitaria Pisana, Pisa, Italy; 3 Division of Medical Oncology, Department of Precision Medicine, University of Study of Campania “L. Vanvitelli”, Naples, Italy; 4 Clinical Oncology Unit, Careggi University Hospital, Florence, Italy; 5 Department of Experimental and Clinical Medicine, University of Florence, Florence, Italy; 6 Department of Oncology and Hematology, University Hospital of Modena, Modena, Italy; 7 Department of Oncology, San Bortolo General Hospital, Azienda ULSS8 Berica, Vicenza, Italy; 8 Medical Oncology Unit, University Hospital and University of Cagliari, Cagliari, Italy; 9 Department of Surgery, Oncology, and Gastroenterology, University of Padova, 35121 Padua, Italy; 10 Department of Oncology, Division of Medical Oncology, Azienda Toscana Nord Ovest, Livorno, Italy; 11 Department of Translational Research and New Technologies in Medicine, University of Pisa, Pisa, Italy; 12 Comprehensive Cancer Center, Fondazione Policlinico Universitario Agostino Gemelli IRCCS, Rome, Italy; 13 Medical Oncology Department, Nuovo Ospedale-Santo Stefano, Prato, Italy; 14 Department of Medical Oncology, Università Vita-Salute, San Raffaele Hospital IRCCS, 20019 Milan, Italy; 15 Department of Medical Oncology, Fondazione IRCCS Istituto Nazionale dei Tumori, Milan, Italy; 16 Medical Oncology 3, Istituto Oncologico Veneto IOV-IRCCS, Padua, Italy

**Keywords:** Gastric cancer, Gastro-esophageal junction adenocarcinoma, Perioperative chemotherapy, FLOT, Microsatellite instability status

## Abstract

**Background:**

FLOT regimen is the standard perioperative treatment in Western countries for patients with locally advanced gastric (GC) or gastroesophageal junction cancer (GEJC). High microsatellite instability (MSI-H) and Mismatch Repair deficient (dMMR) demonstrated a favorable prognostic role and a concomitant negative predictive impact on the benefit of perioperative 5-fluorouracil-based doublets; however, its role in pts receiving FLOT chemotherapy is still unclear.

**Methods:**

This is a retrospective, multicenter observational study of 265 pts with GC/GEJC treated with perioperative FLOT regimen in 11 Italian oncology centers between January 2017 to December 2021 and analyzed for microsatellite status.

**Results:**

The MSI-H phenotype was found in 27 (10.2%) of 265 analyzed tumors. Compared to microsatellite stable (MSS) and Mismatch Repair proficient (pMMR) cases, MSI-H/dMMR were more frequently female (48.1% vs. 27.3%, *p* = 0.0424), elderly pts (age > 70 years, 44.4% vs. 13.4%, *p* = 0.0003), Laurens’s intestinal type (62.5% vs. 36.1%, *p* = 0.02) and pts with a primary location tumor in the antrum (37 vs. 14.3%, *p* = 0.0004). A statistically significant difference in the rate of pathologically negative lymph node emerged (63% vs 30.7%, *p* = 0.0018).

Compared to the MSS/pMMR tumor population, the MSI-H/dMMR subgroup had a better DFS (median not reached [NR] vs. 19.5 [15.59–23.59] mos, *p* = 0.031) and OS (median NR vs. 34.84 [26.68–47.60] mos, *p* = 0.0316).

**Conclusions:**

These real-world data confirm that FLOT treatment is effective in daily clinical practice for locally advanced GC/GEJC, also in the MSI-H/dMMR subgroup. It also showed a higher rate of nodal status downstaging and a better outcome of MSI-H/dMMR pts in comparison to MSS/pMMR.

## Introduction

Globally, GC and GEJC represent a leading cause of cancer-related morbidity and mortality, ranking fifth place in incidence and fourth in mortality overall (Sung et al. 2021).

Surgery is the only potentially curative treatment for resectable T2-T4 or nodal-positive GC/GEJC, yet only around 40% of pts are candidates for resection, and the majority of these pts benefit from a multimodality approach (Lordick et al. 2022). Despite the improvement of multimodal treatments combining chemotherapy and chemoradiation, the 5-year OS is less than 30%, and in the metastatic setting, the prognosis remains poor with a 1-year median OS (Marano et al. 2016).

Recently, in the German phase II/III FLOT4 trial, Al-Batran and colleagues found that perioperative therapy with docetaxel-based triplet FLOT (fluorouracil [5-FU]/leucovorin, oxaliplatin, and docetaxel; four pre- and four postoperative cycles) was associated with improved OS over ECF/ECX (epirubicin and cisplatin plus either 5-FU or capecitabine; three pre- and three postoperative cycles) in pts with resectable locally advanced GC or GEJC. In the FLOT group, the median OS was 50 months compared to 35 months in the ECF/ECX group (HR 0.77, *p* = 0.12). In addition, FLOT surpassed ECF/ECX in secondary endpoints, such as DFS (median 30 vs. 18 months) and rate of R0 resections (85% vs. 78%). In the subgroup analysis, the superiority of FLOT was confirmed regardless of age, presence of signet-ring cell histology, tumor location, or clinical T or N stage (Al-Batran et al. 2019). Based on the OS benefit reported in FLOT4, the FLOT regimen became the new standard of care for pts who can tolerate triplet chemotherapy.

In the absence of validated biomarkers potentially capable to identify pts eligible for adjuvant/perioperative chemotherapy or surgery alone, current treatment decisions for resectable GC/GEJC are currently based on clinical and pathological staging. However, in the last years, MSI-H/dMMR status has emerged as a favorable prognostic factor leading to prolonged survival in analyses of randomized clinical trials, MAGIC and CLASSIC studies (Choi et al. 2019). In a secondary analysis of the MAGIC trial, Smyth and colleagues also identified MSI-H/dMMR as a negative predictive factor of the efficacy of perioperative treatments. In fact, while the positive prognostic impact of the MSI-H/dMMR status was confirmed in pts undergoing surgery alone when compared to MSS/pMMR (median OS: NR versus 20.5 months [HR 0.42; 95% CI 0.15–1.15; *p* = 0.09], in the perioperative chemotherapy arm, median OS was significantly shorter in MSI-H tumor versus the MSS/pMMR group, 9.6 vs. 19.5 months (HR 2.18; 95% CI 1.08–4.42; *p* = 0.03) (Smyth et al. 2017). More recently, an individual patient meta-analysis pooling data from the CLASSIC and MAGIC trials together with the ARTIST and ITACA-S trials, which both compared different multimodal treatment strategies in curative GC/GEJC, confirmed the powerful positive prognostic effect of MSI-H/dMMR in surgically resected GC and GEJC pts and the lack of benefit of perioperative or adjuvant chemotherapy after surgery in this molecularly selected subgroup (Pietrantonio et al. 2019).

Notably, studies questioning the role of chemotherapy in MSI-H/dMMR GC/GEJC did not include taxane-containing regimens, such as FLOT, which is now the standard of care for medically fit pts with stage II-III GC and GEJC. However, data from a small number of MSI-H/dMMR pts treated with FLOT in the phase II DANTE trial (NCT03421288) demonstrated a better response rate than historical with platinum-5-FU (Al-Batran, et al. 2021).

The primary aim of this study is to investigate the real-world efficacy of FLOT and to describe the histopathological features and clinical outcomes of the MSI-H/dMMR subgroup population.

## Methods

### Study design and patient population

In this observational, retrospective, multicenter study, we collected data from locally advanced GC or GEJC pts treated as clinical practice with perioperative FLOT in 11 Italian Oncology Units from January 2017 to December 2021.

Selection criteria were to have received perioperative chemotherapy with FLOT and the availability of microsatellite status and survival outcome data. All pts had locally advanced tumors, defined as cT2 or greater, lymph node-positive (N +), or both.

Our clinical dataset comprised a total of 265 pts with adequate clinical information. Detailed clinicopathologic features were collected for each patient.

Anonymized data were collected for this observational, retrospective and non-interventional study.

### Statistical analysis

The retrospective analysis of clinical data was performed using a prospective database. The chi-squared or Fisher’s test were used to compare clinicopathologic features according to microsatellite mutational status (MSI-H/dMMR vs. MSS/pMMR). Pathological response was evaluated using the Mandard tumor regression grading (TRG) system (Mandard et al. 1994).

OS was calculated from the time of initial diagnosis until death due to any cause. Progression-free survival (PFS) was calculated from the time of initial diagnosis until progression/relapse (distant and/or local), death, or last follow-up. DFS was calculated from the time of surgery to progression/relapse (distant and/or local), death, or last follow-up. OS, PFS, and DFS analyses were performed on the overall population according to the Kaplan–Meier method, and survival curves were compared using the log-rank test. Statistical significance was set at *p* = 0.05 for a bilateral test. The correlation between mutational status and clinicopathologic characteristics with OS was first assessed in univariate analyses. The Cox proportional hazards model was used in the multivariate analysis, which included all the covariates that significantly correlated with OS in the univariate analysis (cut-off, *p* < 0.05).

No formal sample size estimation and power calculation were made for this retrospective observational study.

All statistical analyses were performed using R Statistical Software (version 4.1.2; R Foundation for Statistical Computing, Vienna, Austria).

## Results

### Clinical features and perioperative treatment administration

Baseline characteristics of the 265 pts actually enrolled, major tumor features and clinical course according to MSI-H/dMMR phenotype are summarized in Table 1.

**Table 1:** Baseline patient, tumor, and perioperative treatment characteristics

Characteristic		Total 265 *n*. (%)	MSS/pMMR 238 *n*. (%)	MSI-H/dMMR 27 *n*. (%)	*p* value
Sex	Male	187 (70.6)	173 (72.7)	14 (51.9)	0.0424
	Female	78 (29.4)	65 (27.3)	13 (48.1)	
Age, years	< 70	221 (83.4)	206 (86.6)	15 (55.6)	0.0003
	≥ 70	44 (16.6)	32 (13.4)	12 (44.4)	
ECOG PS	0	218 (82.3)	200 (84.0)	18 (66.7)	0.0338
	1	47 (17.7)	38 (16.0)	9 (33.3)	
Stage at diagnosis	I-II	71 (26.8)	65 (27.3)	6 (22.2)	0.6531
	III	194 (73.2)	173 (72.7)	21 (77.8)	
cN + at diagnosis	Yes	202 (76.2)	183 (76.9)	19 (70.4)	0.4763
Primary location	Antrum	44 (16.6)	34 (14.3)	10 (37.0)	0.0004
	Body	87 (32.8)	75 (31.5)	12 (44.5)	
	GEJ	134 (50.6)	129 (54.2)	5 (18.5)	
Lauren Classification	Intestinal	80 (30.2)	65 (27.3)	15 (55.5)	0.0201
	Mixed	22 (8.3)	19 (8.0)	3 (11.1)	
	Diffuse	102 (38.5)	96 (40.3)	6 (22)	
	Missing	61 (23.0)	58 (24.4)	3 (11.1)	
Grading	G1	7 (2.6)	7 (3.0)	0 (0.0)	1.00
	G2-G3	212 (80.0)	190 (79.8)	22 (81.5)	
	Gx	46 (17.4)	41 (17.2)	5 (18.5)	
HER 2 status	3 + , 2 + Fish amp	22 (8.3)	22 (9.2)	0 (0.0)	0.2386
	Negative	225 (94.9)	198 (83.2)	27 (100.0)	
	Missing	18 (6.8)	14 (5.9)	4 (14.8)	
Preoperative chemotherapy	Completed (4 cycles)	254 (95.8)	227 (95.4)	27 (100.0)	0.6101
	Incomplete (< 4 cycles)	11 (4.2)	11 (4.6)	0 (0.0)	
Surgery	Yes	252 (95.1)	225 (94.5)	27 (100.0)	0.3739
Post-operative chemotherapy	Completed (4 cycles)	142 (53.6)	126 (52.9)	16 (59.25)	0.0787
	Incomplete ($<$ 4 cycles)	44 (16.6)	43 (18)	1 (3.7)	
Received all FLOT without dose reduction	Yes	124 (46.8)	109 (45.8)	15 (55.6)	0.4476

*ECOG PS* Eastern Cooperative Oncology Group Performance Status

The median age was 62 years (range 37–81 years). Seventy-one percent (*n* = 187) of the pts were male and 29% (*n* = 78) female. All pts were in good clinical condition, with an ECOG (Easter Cooperative Oncology Group) performance status (PS) of 0 in the majority of the study population (82.3%, *n* = 218).

Tumors were mainly located in the gastrointestinal junction (50.6%, *n* = 134), corpus (32.8%, *n* = 87), and antrum (16.6%, *n* = 44). According to the Lauren classification, a diffuse-type adenocarcinoma was reported in 38.4% (*n* = 102), intestinal in 30.2% (*n* = 80), and mixed in 8.3% (*n* = 22). The Lauren subtype was not specified in 23% of cases.

The MSI-H/dMMR phenotype was identified in 27 of the 265 tumors analyzed (10.2%), while there were 22 non-overlapping HER-2 positive cases (8.3%).

In comparison to MSS/pMMR cases, MSI-H/dMMR were more prevalent in females (48.1% vs. 27.3%, *p* = 0.0424), elderly pts (age ≥ 70 years, 44.4% vs. 13.4%, *p* = 0.0003), Laurens’s intestinal type (55.5% vs. 22% diffuse type, *p* = 0.02) and in pts with a primary tumor location in the antrum (37% vs. 14.3%, *p* = 0.0004).

There was no significant correlation between MSI-H/dMMR and stage (*p* = 0.65) or grading (*p* = 1). At the time of initial diagnosis, the majority of pts were in stage III (73.2%, *n* = 194).

Surgical resection was performed in 252 of the 265 pts (95.1%), and 186 pts started adjuvant chemotherapy (73.8%), with 142 of these (56.3%) completing the entire treatment plan with 4 cycles of post-operative FLOT.

### Pathological findings

Histopathological characteristics according to the MSI-H/dMMR phenotype are listed in Table 2.

**Table 2:** Histopathological findings

Characteristic		Total 252 *n*. (%)	MSS/pMMR 225 *n*. (%)	MSI-H/dMMR 27 *n*. (%)	*p* value
Post-operative pathological stage	ypT0	17 (6.7)	16 (7.1)	1 (3.7)	1.00
	ypN0	86 (34.1)	69 (30.7)	17 (63.0)	0.0018
	ypT0N0	14 (5.5)	13 (5.7)	1 (3.7)	0.47
Tumor regression grade	Regression complete (TRG1)^a^	19 (7.5)	18 (8)	1 (3.7)	0.5418
	Subtotal (TRG2)^a^	12 (4.8)	11 (4.8)	1 (3.7)	
	Partial (TRG3)^a^	49 (19.4)	46 (20.4)	3 (11.1)	
	No Regression (TRG4-5)^a^	97 (38.5)	84 (37.3)	13 (48.1)	
	Missing	75 (29.8)	66 (29.3)	9 (33.3)	
Resection	R0	226 (89.7)	200 (88.9)	26 (96.3)	0.3281
	R1	23 (9.1)	22 (9.8)	1 (3.7)	
	R2	3 (1.2)	3 (1.3)	0	

^a^ACCORDING to the Mandard classification

*TRG* Tumor regression grade

All surgical margins were negative in 226 specimens of the 252 tumors resected (R0 resection, 89.7%), whereas, in the case of 23 specimens (9.1%), tumor was microscopically present at the distal surgical margins. During surgery, distant metastases emerged as intraoperative finding only in three cases (1.2%).

Fourteen specimens (5.5% of the resected population) had no residual tumor following neoadjuvant therapy and these pts were evaluated at stage ypT0N0.

No major differences in clinical N + status were detected by MSI groups at baseline. However, a statistically and clinically significant difference in the surgical specimens’ rate of negative lymph nodes was observed: 63% in MSI-H/dMMR cases, compared to 30.7% in MSS/pMMR specimens (*p* = 0.0018).

Mandard classification data on tumor regression was available for 159 of the 225 MSS tumors and 18 of the 27 MSI-H/dMMR tumors. In the MSS/pMMR group, histological complete remission was observed (TRG1) in 18 pts (11.3%), subtotal histological regression in 11 pts (TRG2, 6.9%), and partial regression was observed in 46 pts (TRG2-3, 28.9%); 84 pts TRG4-5, 52.8%) had a poor response. One patient from among the 18 MSI-H/dMMR pts had a histopathological complete response, whereas 13 pts (72.2%) did not achieve a relevant histological regression.

### Clinical outcomes

At the time of analysis, 138 out of 252 resected pts (54.8%) had disease progression, with 5 of them (3.6%) presenting isolated local recurrence and 133 (96.4%) distant recurrence.

The rate of recurrence following R0 or R1 resection was 57.3% (*n* = 129) in the MSS /pMMR cohort and 33.3% (*n* = 9) in the MSI-H/dMMR population (*p* = 0.0638). The sites of distant recurrence were mainly peritoneal carcinomatosis (40.6%, *n* = 56), nodes (34.8%, *n* = 48), and liver (21.7%, *n* = 30), with no statistically significant difference between the two groups in terms of recurrence site. Pattern of recurrence and treatments in metastatic settings according to MSI-H/dMMR phenotype are listed in Table 3.

**Table 3:** Pattern of recurrence and treatments in metastatic setting

Characteristic		Total 138 *n*. (%)	MSS/pMMR 129 *n*. (%)	MSI-H/dMMR 9 *n*. (%)	*p* value
Time to metastases	Synchronous	38 (27.5)	37 (28.7)	1 (11.1)	0.4439
	Metachronous	100 (72.5)	92 (71.3)	8 (88.9)	
Metastatic disease resection	Yes	16 (11.6)	14 (10.9)	2 (22.2)	0.2797
Sites of metastases	Liver yes	30 (21.7)	28 (21.7)	2 (22.2)	1.00
	Nodes yes	48 (34.8)	44 (34.1)	4 (44.4)	0.7190
	Lung yes	10 (7.2)	10 (7.8)	0 (0.0)	1.00
	Peritoneum yes	56 (40.6)	55 (42.6)	1 (11.1)	0.0831
	Bone yes	7 (5.1)	6 (4.7)	1 (11.1)	0.3831
	Brain yes	7 (5.1)	7 (5.4)	0 (0.0)	1.00
	Other yes	10 (7.2)	9 (7.0)	1 (11.1)	0.5026
	Only local yes	5 (3.6)	4 (3.1)	1 (11.1)	0.2899
Firts-line therapy	Yes	92 (66.7)	85 (65.9)	7 (77.8)	0.71
	Platin-based therapy	36 (39.1)	36 (42.3)	0 (0.0)	
	Taxane-based therapy	18 (19.6)	17 (20.0)	1 (14.2)	
	Irinotecan based therapy	34 (37.0)	31 (36.5)	3 (42.9)	
	Others	4 (4.3)	1 (1.2)	3 (42.9)	
Second-line therapy	Yes	45 (32.6)	42 (32.5)	3 (33.3)	1.00
	Taxane-based therapy	29 (64.5)	28 (66.6)	1 (33.3)	
	Irinotecan-based therapy	12 (26.7)	12 (28.6)	0 (0.0)	
	Immunotherapy	2 (4.4)	0 (0.0)	2 (66.7)	
	Others	2 (4.4)	2 (4.8)	0 (0.0)	
	Yes	14 (10.1)	13 (10.7)	1 (11.1)	1.00
Third-line therapy	Platin-based doublet	(14.3) 2	2 (16.4)	0 (0.0)	
	Irinotecan-based therapy	3 (21.4)	3 (23.0)	0 (0.0)	
	Immunotherapy	4 (28.6	4 (30.8)	0 (0.0)	
	Others (Triflurina-Tipiracil)	5 (35.7)	4 (30.8)	1 (100.0)	

In the metastatic setting, surgery with curative intent was attempted in 16 out of 138 (11.6%) pts.

No statistically significant difference between the two groups in terms of the number of post-progression treatment lines was observed. In fact, in the MSS/pMMR cohort, 88 pts underwent first-line treatments, compared to 7 pts in the MSI-H/dMMR cohort (respectively, 65.9% vs. 77.8%, *p* = 0.71).

**Fig. 1:**
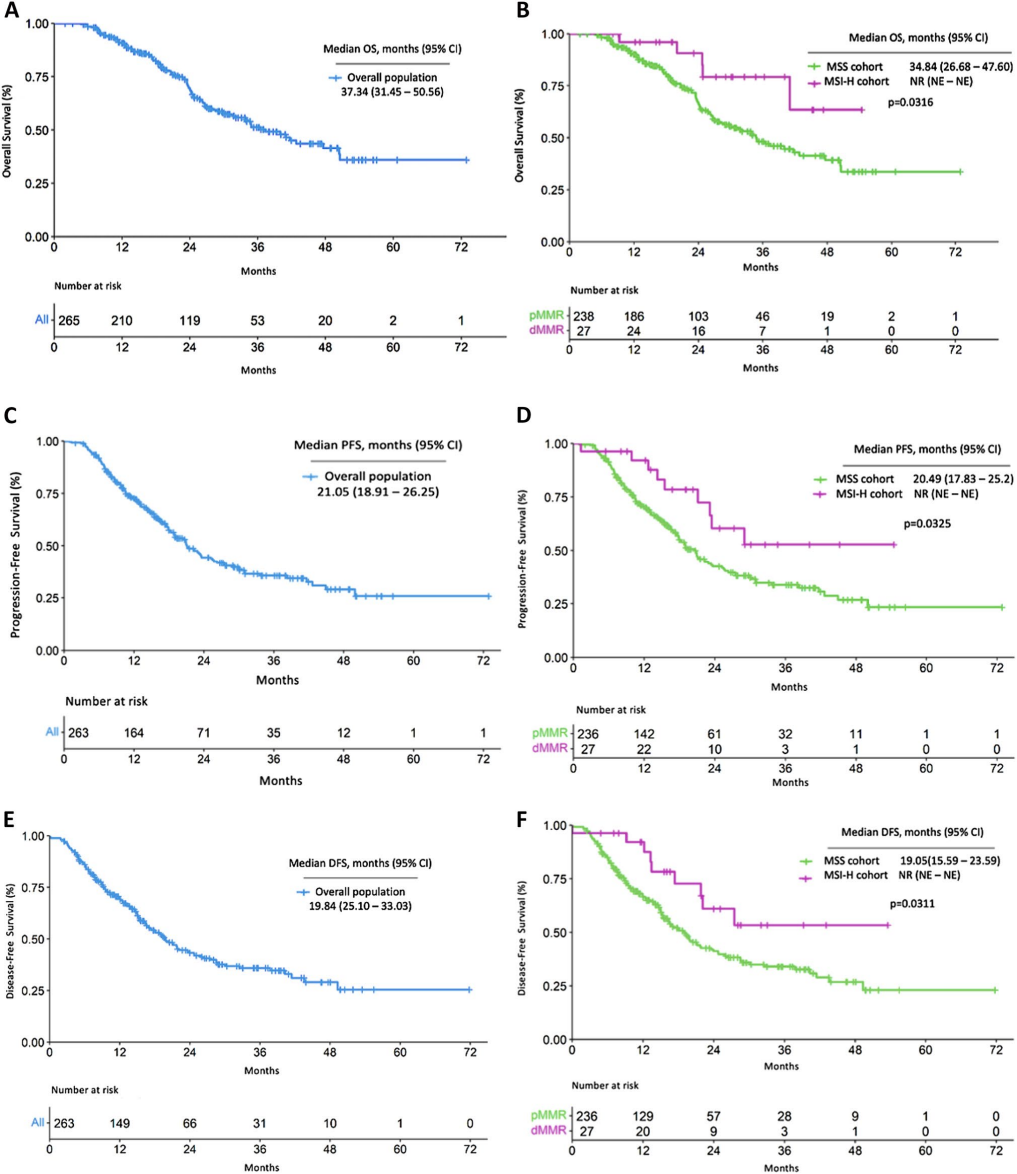
Efficacy. *OS* Overall Survival, *PFS* Progression Free Survival, *DFS* Disease-Free Survival, 95% *CI* confidence interval, **A** Kaplan–Meier curve for OS in the overall population. **B** Kaplan–Meier curves for OS in MSS/pMMR and MSI-H/dMMR cohorts.**C** Kaplan–Meier curve for PFS in the overall population). **D** Kaplan–Meier curves for PFS in MSS/pMMR and MSI-H/dMMR cohorts.**E** Kaplan–Meier curve for DFS in the overall population. **F** Kaplan–Meier curves for DFS in MSS/pMMR and MSI-H/dMMR cohorts

At the time of data collection, with a median follow-up period of 30.86 months, median OS of the whole population was 37.34 months (95% CI 31.45–50.56). The median OS for MSS/pMMR pts was 34.84 months (95% CI 28.68–47.60), whereas the median OS for MSI-H/dMMR group was not reached, (*p* = 0.0316), Fig. 1A, B.

In MSI-H/dMMR vs. MSS/pMMR comparison, the 2-year OS was 90.7% (95% CI 75—100%) vs. 66.5% (95% CI: 59.9—73.8%), *p* = 0.0301, yet only 2 vs. 63 events were observed at that time-point.

The median PFS based on 138 events was 21.05 months (95% CI 18.91–26.25) for the global population. The median PFS for MSS/pMMR pts was 20.49 months (95% CI 17.83–25.2), whereas the median PFS was not reached for MSI-H/dMMR pts (*p* = 0.0325), Fig. 1C, D.

Similar results were observed for DFS in the whole cohort, the median DFS based on 138 events was 19.84 months (95% CI 16.71–24.77). The median DFS for MSS/pMMR pts was 19.05 months (95% CI 15.59–23.59), whereas was not reached in the MSI-H/dMMR group (*p* = 0.0311), Fig. 1E, 1F.

We performed additional survival analyses to identify characteristics that may have affected outcomes in this patient population.

As expected, univariate analyses for OS showed that positive resection margins and diagnosis of metastatic disease had a negative impact, while complete neoadjuvant treatment, microsatellite instability status, intestinal-histotype, negative lymph node status (ypN0) and no residual tumor in the surgical specimen (ypT0) were positive prognostic factors.

In multivariate analysis, diagnosis of metastatic disease [Hazard Ratio (HR) 10.40, 95% CI 4.42–24.47, *p* = 0.0001 and radicality of resection (HR 8.34, 95% CI 1.89—36.83, *p* = 0.0052) also remained independently associated with OS, Table 4.

**Table 4:** Univariate and multivariate models for overall survival

Variables	Category	Univariate			Multivariate		
		HR	95% CI	*P* value	HR	95% CI	*P* value
Gender (ref: Female)	Male	1.14	0.74–1.77	0.5439			
Age (ref: < 70 years)	≥ 70 years	0.70	0.39–1.26	0.2396			
ECOG PS (ref: 0)	1	1.21	0.76–1.95	0.4224			
Primary location (ref: Body)	GEJ	1.20	0.78–1.84	0.4117			
	Antro	0.86	0.46–1.57	0.5954			
Histological type- Lauren classification (ref: Intestinal)	Diffuse	2.09	1.28–3.39	0.0031*	1.61	0.93–2.80	0.0915
	Mixed	2.14	1.03–4.47	0.0422*	1.61	0.68–3.82	0.2831
	Other	1.10	0.59–2.04	0.7656	1.35	0.68–2.69	0.3915
HER2 status (ref: Positive)	Negative	1.09	0.51–2.35	0.8255			
Stage at diagnosis (ref: I-II)	III	1.21	0.77–1.91	0.4008			
Neoadjuvant chemotherapy (ref:complete)	Incomplete	2.53	1.23–5.22	0.0120*	1.86	0.79–4.40	0.1558
Adjuvant chemotherapy (ref:complete)	Incomplete	1.17	0.66–2.07	0.5874			
ypT0 (ref: 0)	Not equal to 0	4.34	1.06–17.71	0.0407*	1.85	0.43–7.95	0.4084
ypN0 (ref:0)	Not equal to 0	2.28	1.37–3.80	0.0015*	0.98	0.57–1.71	0.9496
Resection (ref: R0)	R1	2.43	1.34–4.40	0.0034*	1.64	0.90–3.00	0.1092
	R2	18.60	4.29–80.59	< 0.0001*	8.34	1.89–36.83	0.0052*
Metastatic disease (ref: No)	Yes	12.38	5.74–26.71	< 0.0001*	10.40	4.42–24.47	< 0.0001*
Microsatellite status (ref: MSI-H/dMMR)	MSS/pMMR	2.59	1.05–6.36	0.0384*	2.01	0.78–5.19	0.15

95% *CI* confidence interval, *ECOG PS* Eastern Cooperative Oncology Group performance status, HR hazard ratio, *Ref* reference

## Discussion

Since the publication of the FLOT4-AIO study, perioperative FLOT has been regarded as the new standard of care for pts with locally advanced GC or GEJC (Al-Batran et al. 2019). As is known, data reported outside of clinical trials are essential for determining whether the results can be applied to a real-world setting.

Our retrospective study is the first and largest to investigate the real-world efficacy of perioperative FLOT in pts with GC and GEJC and to describe histopathological features and clinical outcomes according to MSI status.

The present findings support the reproducibility of FLOT efficacy in a subset of real-world pts, who were not selected as favorably as those in the clinical trial.

Data from the FLOT4 trial confirmed those from the MAGIC and FNCLCC-FFCD studies concerning suboptimal adherence to post-operative adjuvant CT, with less than 50% of pts completing all scheduled cycles (Al-Batran et al. 2019; Cunningham et al. 2006; Ychou et al. 2011). In our population, however, a higher proportion of pts (56.3%) completed all the post-operative phase with a further 4 cycles of FLOT (142 out of 252 pts who underwent surgery), while an additional 16.6% of the resected pts were able at least to start adjuvant FLOT and to receive 1–3 cycles. In order, to further underline the feasibility of the FLOT regimen, our data are gathered from the management of 11 Oncology Units distributed throughout Italy, and not just from a single referral center.

Since the pivotal publication of the molecular classification of gastric adenocarcinoma in 2014, (N., Comprehensive molecular characterization of gastric adenocarcinoma 2014) the prognostic and also the predictive significance of MSI-H/dMMR status in GC/GEJC has been brought into focus. In a recent meta-analysis representing the largest analyzed data set with more than 18,000 pts (including Caucasian and Asian cohorts), the rate of MSI-H/dMMR tumors was 9.2%, (Polom et al. 2018) which is consistent with our finding of 10.4%.

In our study, consistent with previous findings, MSI-H/dMMR GC was associated with older age (> 70 years), female gender, and tumoral site in the lower gastric body, particularly the antrum. Moreover, the majority of MSI-H/dMMR pts (62.5%) had an intestinal subtype. In concordance with other reports, however, we also found MSI-H/dMMR tumors among diffuse and mixed-type tumors, in which the beneficial outcome in comparison to MSS tumors was questioned by Marrelli et colleagues (Polom et al. 2018; Zubarayev et al. 2019; Marrelli et al. 2016). In contrast to the prevalent conception that MSI-H/dMMR phenotype in GC is restricted to intestinal-type tumors, our data provide further evidence that restricting microsatellite status analysis (also with regard to Lynch syndrome diagnoses) to intestinal-type GC may not be adequate.

Our analyses did not reveal that MSI-H/dMMR was enriched in early-stage cancers. As has been suggested, (An et al. 2012) this could be explained by the fact that the pts enrolled in our cohort were all candidates for the FLOT regimen and, therefore, presented with more locally advanced disease. Indeed, only 4% of our population was diagnosed with stage I and only 22.8% had stage II.

Although the positive prognostic value of MSI-H/dMMR in localized GC and GEJC is consistent across studies, evidence of MSI-H/dMMR as a negative predictor of the efficacy of neoadjuvant or adjuvant chemotherapy remains questionable. Indeed, no systematic data on the clinical outcomes of MSI-H/dMMR cancer treated with the perioperative FLOT regimen have been reported to date. Therefore, translational analyses of the FLOT4 trial dataset will be of paramount relevance to draw conclusions on MSI-H/dMMR and other potential biomarkers.

According to a recent metanalysis by Polom and colleagues, (Polom et al. 2018) pts with MSI-H /dMMR tumors have a better outcome than those with MSS/pMMR tumors. However, the benefits of perioperative or adjuvant chemotherapy are unclear, based on the exploratory analyses of recent phase III trials (Choi et al. 2019; Smyth et al. 2017)(Boyer et al. 2023).

In a recent meta-analysis, Pietrantonio and colleagues evaluated the prognostic and predictive role of MSI in Asian and Western pts with resectable GC, using data from 1556 pts enrolled in four international phase III trials of perioperative, neoadjuvant and adjuvant treatment (MAGIC, CLASSIC, ITACA-S, and ARTIST trials). (Pietrantonio et al. 2019) The authors demonstrated that MSI-H/dMMR status was associated with a better DFS and OS compared to MSS/pMMR disease, confirming the positive prognostic role of this biomarker. In addition, MSI-H/dMMR was associated with no benefit from chemotherapy when compared to surgery alone, suggesting that pts could be selected to undergo perioperative or adjuvant treatment based on the tumor’s microsatellite status at diagnosis. It should be noted, however, that taxane-based regimens were not included in these meta-analyses and the role of MSI-H/dMMR status in pts treated with taxane combinations is poorly explored in the literature. Haag and colleagues found no evidence of a negative effect of perioperative FLOT in the MSI-H/dMMR cohort (Haag et al. 2019). In addition, Kohlruss et al., who unfortunately did not report on the applied chemotherapy regimens, found that MSI-H/dMMR status was not associated with worse OS in pts treated with neoadjuvant chemotherapy (HR 0.54, 95% CI: 0–26 – 1.09) (Kohlruss et al. 2019). In accordance with these findings, our results demonstrated that the MSI-H/dMMR group had a statistically superior clinical outcome, although in multivariate analysis MSI-H/MMRd is not independently associated with OS. The median OS for MSS/pMMR pts was 34.84 months (95% CI 26.68–47.60), whereas was not reached for MSI-H/dMMR group (*p* = 0.0316). The median PFS for MSS/pMMR pts was 20.49 months (95% CI 17.83–25.2), whereas the median PFS was not reached in the MSI-H/dMMR group (*p* = 0.0325). Furthermore, there was a statistically significant difference in the rate of negative lymph node status between the MSS/pMMR and MSI-H/dMMR cohort (30.7% vs. 63%, *p* = 0.0018), and indeed nodal positivity is a well-known negative prognostic factor for relapse and OS in multiple studies (Smyth et al. 2016; Tang et al. 2021; Tran-Minh et al. 2021).

Undoubtedly, a comparison of radiological lymph nodes status before chemotherapy and pathological lymph nodes status after chemotherapy is not methodologically correct, as CT-scan staging may under- or overestimate nodal positivity. However, in our opinion this bias should have affected both the MSS/pMMR and MSI-H/dMMR group evaluation, as there is no solid evidence of different CT-scan sensitivity and specificity for nodal positivity according to MSI status. Therefore, despite caution, we believe that our finding on the different impacts on nodal downstaging might be taken into account.

Furthermore, consistently with previous findings, we observed a poor histological response to neoadjuvant chemotherapy in the majority of MSI-H/dMMR tumors (72.2%, *n* = 13), rebutting the therapy’s potential benefit. Even if the rate of histologically poor responders did not formally differ between the MSS/pMMR and MSI-H/dMMR cohorts, there was non-responding tumor enrichment in the MSI-H/dMMR subgroup. Despite these findings, the outcome of pts in the MSI-H/dMMR cohort tended to be better than in the MSS/pMMR cohort, suggesting that histopathological response is not a good predictor of survival in MSI-H/dMMR (Haag et al. 2019) Still, we cannot completely rule out the benefit of cytotoxic treatment, as suggested by Giampieri in the metastatic setting, (Giampieri et al. 2017) in which the microenvironment shifts toward a tumor-suppressive milieu and hence promotes an immunological response.

Even with the limited statistical power due to the low prevalence of MSI-H/dMMR, in line with the literature, our data showed a 20% quantitative difference between the MSS/pMMR and MSI-H/dMMR subgroups in the relapse rate after R0 or R1 resections. This suggests that the prognostic impact of MSI-H/dMMR was maintained even in pts treated with the FLOT regimen and at the very least excludes a detrimental effect, as previously reported (Smyth et al. 2017).

Some other limitations of our study should also be addressed. First, the present analysis is certainly limited by its non-randomized nature. Pts were not randomized to receive perioperative chemotherapy versus straight-up surgery versus different multimodal protocols. This means that, while we can address the prognostic value of MSI-H/dMMR status on FLOT efficacy, we cannot address the predictive value because we lack a non-FLOT-treated group. Second, the recurrence rate may be underestimated due to incomplete follow-up data in some cases, as is the be in retrospective multicenter studies. Thirdly, the median follow-up period (30.86 months) was relatively short in our study, resulting in a relatively low number of events and that certainly limits our data’s statistical power. In addition, while surgery in stage IV GC is not a standard procedure, survival might be slightly affected by 11.6% of oligometastatic pts receiving resection after multidisciplinary team discussion. Despite these limitations, we believe that our study may be relevant for better refining treatments strategies in locally advanced GC and GEJC, confirming MSI-H/dMMR as a robust prognostic marker in these patients. Indeed, new perspectives are rapidly approaching and influencing the clinical role of MSI status. As anti-programmed death 1 antibody are associated with response rates greater than 50% in advanced MSI-H GC (Shitara et al. 2018; Fuchs et al. 2018), data from ongoing trials evaluating the administration of checkpoint inhibitors in MSI-H/dMMR GC pts will resolve the dilemma of whether or not to administer FLOT to MSI-H/dMMR pts. Recent clinical trials are investigating the role of checkpoint inhibitors and/or target therapy in addition to the perioperative treatments, based on results in metastatic settings to improve pCR rate and survival outcomes (Catanese and Lordick 2021). Active studies that are incorporating immunotherapy with perioperative chemotherapy include KEYNOTE 585 (ClinicalTrials.gov: NCT03221426) with pembrolizumab/placebo and the DANTE/FLOT8 trial (ClinicalTrials.gov: NCT03421288) which is assessing the addition of atezolizumab to perioperative FLOT. Further insights into biomarkers of perioperative chemo-immunotherapy (FLOT-avelumab) will be elucidated by the phase II ICONIC study (ClinicalTrials.gov: NCT03399071). Lenvatinib, an orally available multi-kinase inhibitor, is also being investigated in combination with pembrolizumab as a perioperative treatment (ClinicalTrials.gov: NCT04745988). Even if all these trials were not specifically designed for MSI-H/dMMR status, they will undoubtedly provide retrospective evidence on this subset of pts.

More importantly, the primary results of the phase II NEONIPIGA trial (NCT04006262) showed a high pathologic complete response rate (59%) with nivolumab/ipilimumab neoadjuvant therapy in pts with MSI-H localized GC or GEJC adenocarcinoma (Andre et al. 2022). Also, the ongoing proof-of-concept INFINITY study (NCT04817826) is specifically designed for MSI-H resectable GC/GEJC pts, and investigates the role of the durvalumab + tremelimumab combination as a neoadjuvant potentially definitive treatment (avoiding surgery in the case of complete clinical response) (Raimondi et al. 2021).

In addition, the IMHOTEP trial is also currently evaluating pembrolizumab in the perioperative setting in MSI-H tumors (Coutzac et al. 2022).

## Conclusions

While we await the results of neoadjuvant immunotherapy treatment of MSI-H/dMMR GC/GEJC, our real-world study reassures the role of FLOT chemotherapy in this population, in which a good prognosis has been observed.

Considering the benefit of node downstaging observed in MSI-H/dMMR tumors in our analysis, a reasonable option nowadays could be to discuss in the multidisciplinary tumor board and to propose FLOT perioperative therapy only in pts with clinical positive nodal status (cN +), and to offer upfront surgery to pts with an MSI-H/dMMR GC/GEJC clinically negative nodal status (cT2-cT3 N0).

Alternative approaches, like immune checkpoint blockade, should be prospectively investigated in MSI-high GCs according to the clinically and pathologically defined risk of relapse.

## Data Availability

The datasets generated during and/or analysed during the current study are available from the corresponding author on reasonable request.
